# Supplementary material for: Preclinical Assessment of ADAM9-Responsive Mesoporous Silica Nanoparticles for the Treatment of Pancreatic Cancer
Source: Int J Mol Sci. 2023 Jun 27;24(13):10704. doi: 10.3390/ijms241310704 (PMC10341742; doi:10.3390/ijms241310704)
Supplement: Supplementary file 1 [file ijms-24-10704-s001.zip › ijms-2441739-supplementary.pdf]

International Journal of
Molecular Sciences

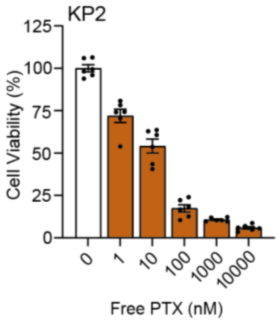

Supplemental Figure S1. Sensitivitiy to free paclitaxel make KP2 cells a suitable *in vivo* model.

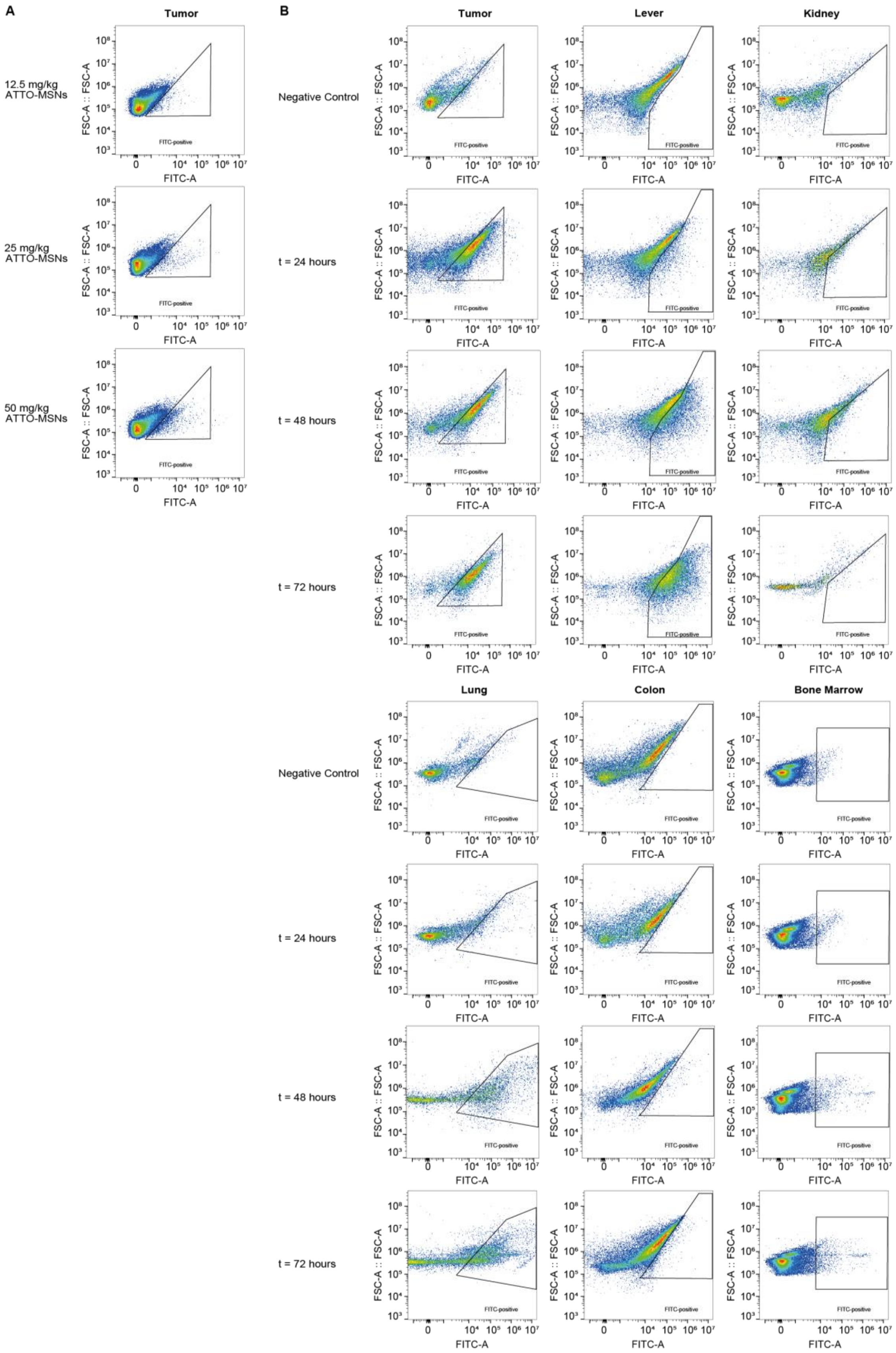

Supplemental Figure S2. Gating Strategy *in vivo* biodistribution of ATTO488-MSNs. **(A)** Optimal dose determination by injection of multiple concentrations of ATTO488-MSNs as measured in excised tumor. **(B)** Accumulation of ATTO488-MSNs in tumor and several major organs at several time points to determine optimal treatment schedule.

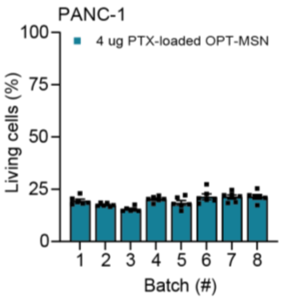

Supplemental Figure S3. OPT-MSNs for *in vivo* tumor toxicity experiment are efficiently loaded with paclitaxel. PANC-1 cells are efficiently killed by low amounts of paclitaxel-loaded OPT-MSNs.
